# Early dystrophin loss is coincident with the transition of compensated cardiac hypertrophy to heart failure

**DOI:** 10.1371/journal.pone.0189469

**Published:** 2017-12-21

**Authors:** Fernanda P. Prado, Daniele O. dos Santos, Valdecir Blefari, Carlos A. Silva, Juliano Machado, Isis do Carmo Kettelhut, Simone G. Ramos, Marcelo Dias Baruffi, Helio C. Salgado, Cibele M. Prado

**Affiliations:** 1 Department of Pathology, School of Medicine of Ribeirão Preto, University of São Paulo, Ribeirão Preto, SP, Brazil; 2 Department of Phisiology, School of Medicine of Ribeirão Preto, University of São Paulo, Ribeirão Preto, SP, Brazil; 3 Department of Biochemistry/Immunology, School of Medicine of Ribeirão Preto, University of São Paulo, Ribeirão Preto, SP, Brazil; 4 Department of Clinical Analysis, Toxicology and Food Science, Faculty of Pharmaceutical Sciences of Ribeirão Preto, University of São Paulo, Ribeirão Preto, SP, Brazil; Niigata Daigaku, JAPAN

## Abstract

Hypertension causes cardiac hypertrophy, one of the most important risk factors for heart failure (HF). Despite the importance of cardiac hypertrophy as a risk factor for the development of HF, not all hypertrophied hearts will ultimately fail. Alterations of cytoskeletal and sarcolemma-associated proteins are considered markers cardiac remodeling during HF. Dystrophin provides mechanical stability to the plasma membrane through its interactions with the actin cytoskeleton and, indirectly, to extracellular matrix proteins. This study was undertaken to evaluate dystrophin and calpain-1 in the transition from compensated cardiac hypertrophy to HF. Wistar rats were subjected to abdominal aorta constriction and killed at 30, 60 and 90 days post surgery (dps). Cardiac function and blood pressure were evaluated. The hearts were collected and Western blotting and immunofluorescence performed for dystrophin, calpain-1, alpha-fodrin and calpastatin. Statistical analyses were performed and considered significant when p<0.05. After 90 dps, 70% of the animals showed hypertrophic hearts (HH) and 30% hypertrophic+dilated hearts (HD). Systolic and diastolic functions were preserved at 30 and 60 dps, however, decreased in the HD group. Blood pressure, cardiomyocyte diameter and collagen content were increased at all time points. Dystrophin expression was lightly increased at 30 and 60 dps and HH group. HD group showed decreased expression of dystrophin and calpastatin and increased expression of calpain-1 and alpha-fodrin fragments. The first signals of dystrophin reduction were observed as early as 60 dps. In conclusion, some hearts present a distinct molecular pattern at an early stage of the disease; this pattern could provide an opportunity to identify these failure-prone hearts during the development of the cardiac disease. We showed that decreased expression of dystrophin and increased expression of calpains are coincident and could work as possible therapeutic targets to prevent heart failure as a consequence of cardiac hypertrophy.

## Introduction

Hypertension causes cardiac hypertrophy, one of the most important risk factors for heart failure (HF). Structural remodeling of the heart as a consequence of systemic hypertension is a compensatory mechanism that initially protects the heart, but a sustained excessive cardiac workload is a major predictor of HF and sudden death [1–4]. In spite of the importance of cardiac hypertrophy as a risk factor for the development of HF, not all hypertrophied hearts will ultimately fail [5–8]. Although fibrosis and myocyte hypertrophy are standard markers for myocardial abnormalities in HF, cytoskeletal and sarcolemma-associated proteins alterations have been described [9–13]. The intracellular cytoskeletal proteins are mechanically linked to the extracellular matrix by the dystrophin-glycoprotein complex (DGC). Dystrophin provides mechanical stability to the plasma membrane through its interactions with the actin cytoskeleton and, indirectly, to extracellular matrix proteins. Dystrophin loss is an important mechanism for destabilizing cardiomyocytes, leading to membrane instability and permeability defects [14,15]. Besides, loss of dystrophin has been linked with end-stage cardiomyopathies and has been proposed as a common pathway for contractile dysfunction in the failing myocardium and progression to HF [13,16,17]. The cleavage of dystrophin and other cytoskeletal and submembranous proteins has been demonstrated by several proteolytic enzymes [18–22]. Importantly, calpain-mediated degradation of dystrophin has been highlighted [13,17,19,23,24].

The mechanisms underlying the development of HF as a consequence of hypertension/cardiac hypertrophy are multiple, complex and not totally understood. This fact underscores the need to differentiate the pathways responsible for the transition from compensated cardiac hypertrophy from those promoting decompensation, dilation, and extreme ventricular remodeling. Additional mechanisms besides those that cause hypertrophy could be recruited during progression from compensated cardiac hypertrophy to HF. In this study, our focus was over dystrophin and the intracellular protease calpain.

## Materials and methods

This study was carried out in strict accordance with the recommendations in the Guide for the Care and Use of Laboratory Animals of the National Institutes of Health. The protocol was approved by the Committee on Ethics in Animal Research of Ribeirão Preto School of Medicine, University of São Paulo, SP, Brazil (Protocol no. 204/2009). Male Wistar rats weighing 150.7±6.34 g were maintained on a 12 h light/dark cycle and housed 3 to 5 rats per cage with free access to food and water. All efforts were made to minimize animal suffering and to decrease the number of animals used.

### Experimental protocol

The animals (n = 90) were randomly divided into two groups: operated group, animals subjected to surgical abdominal aorta stenosis and the sham-operated group as control, animals subjected to similar procedure without banding the aorta. After 30, 60 and 90 days post-surgery (dps), the animals were euthanized and the tissues collected for the morphological or molecular analysis. Briefly, the rats were anesthetized with inhaled isoflurane (1.5%) vaporized in medical O_2_. Under sterile conditions, the abdominal aorta above the renal arteries was exposed through a midline abdominal incision and constricted at the suprarenal level by a 4–0 cotton suture tied around both the aorta and a 0.85 mm diameter blunted probe which was then pulled out, as previously described [25]. A similar procedure was performed for sham group, except without the ligature. At the end of surgery, paracetamol (500 mg/kg) was administered orally for pain relief, 6/6h up to 48h after surgery.

### Evaluation of cardiac structure and function by echocardiography

Systolic and diastolic cardiac function were determined noninvasively by transthoracic echocardiography of anesthetized rats (1.5% isoflurane) using a Vevo 2100 high-resolution imaging system equipped with a 21 (small rats) and a 12 MHz-scan head (larger rats) (VisualSonics, Toronto, Canada) (n = 10-15/group). Echocardiography was performed 30, 60 and 90 dps. Images were analyzed off-line by an investigator blinded to the groups using VisualSonics Cardiac Measurements software. For each parameter, measurements were performed from three to six different cardiac cycles, and the values were averaged. Interventricular septum thickness (IVS), left ventricle internal diameter (LVID), and left ventricle posterior wall thickness (LVPW) were measured in both systole and diastole from long-axis views from the parasternal approach. Fractional shortening (FS) and ejection fraction (EF) were calculated. Diastolic function was assessed using pulsed-wave Doppler imaging of the transmitral filling pattern. Mitral inflow pattern was assessed from the apical 4-chamber view with a sample volume placed at the mitral leaflet tips. Peak velocity during early filling (E) was measured. As E and A diastolic filling waves are often merged in rats, A-wave velocity and E-wave deceleration time were not measured in this study. Tissue Doppler assessments were conducted from the apical 4-chamber view with the sample volume placed at the septal portion of the mitral annular level. The lateral mitral annulus was not assessed due to unreliable visibility and poor angulation. Early mitral annular septal velocity (E') was measured and E/E' ratio calculated.

### Blood pressure

The evaluation of blood pressure was performed in the anesthetized animals (pentobarbital sodium, 40 mg/kg, intraperitoneally) one day after echocardiography at 90 dps (n = 6-8/group). Also, some animals were randomly chosen at 30 and 60 dps for the evaluation of the blood pressure (n = 6-8/group). Systolic, diastolic, and mean arterial blood pressures were recorded through polyethylene catheters positioned in the carotid artery and connected to the pressure transducer Powerlab (AD Instruments, Castle Hill, Australia). The values were recorded during 5–8 minutes after hemodynamic stabilization.

### Harvesting of the hearts

Immediately after blood pressure analysis at each time point, the thoracic cavity was opened, the hearts rapidly removed, rinsed in 0.9% saline solution, blotted and weighed. Both ventricles from each heart were isolated and cut into two fragments by a midi ventricular coronal section. The upper fragment was fixed in phosphate-buffered 10% formalin (pH 7.3) for histological study. From the inferior fragment, a thin slice was cut, embedded in Tissue Tek®, frozen in isopentane chilled in liquid nitrogen and stored at -80°C for immunofluorescence study. The remaining from this fragment was frozen in liquid nitrogen and stored at -80°C for Western blot analysis.

To calculate cardiomyocyte diameter in the left ventricle (n = 6-8/group), hematoxylin and eosin stained sections were used. Since the myocardial fiber has a tubular shape, the smallest axis is a measure perpendicular to the nucleus, no matter the myocardial fiber is sectioned in longitudinal, oblique or transversal manner [26,27]. We measured the axis in a section perpendicular to the longest axis of the myocardial fiber crossing the nucleus. Thirty values were obtained per animal, and the mean value was calculated. To estimate the percentage of fibrosis in the left ventricle (n = 6-8/group), the collagen area was determined in picrosirius red-stained sections. For each heart, ten microscopic fields were randomly selected and perivascular fibrosis areas were excluded from the analyses. The mean value was subsequently calculated. Both quantitative examinations were performed on a medium power light-microscopic field (×400). The measurements were performed with the Leica Qwin software (Leica Imaging Systems Ltd., Cambridge, UK) by an investigator blinded to the group’s identification.

### Western blot analysis

The hearts were collected as described before (n = 5-8/group), the left ventricle walls isolated and homogenized in RIPA buffer with protease inhibitor cocktail (Sigma-Aldrich, Inc., St. Louis, MO, USA). Fifty μg protein/well were resolved on a 5%-16% SDS-Page gels and transferred to PVDF membrane (Amersham Pharmacia Biotech, Amersham, UK). The membranes were blocked with 5% albumin for 2 h and incubated overnight at 4°C with the primary antibodies to dystrophin (1:50, Santa Cruz Biotechnology, Santa Cruz, CA, USA), calpain-1 (1:500, Cell Signaling, Danvers, MA, USA), calpastatin (1:500, Cell Signaling) and alpha-fodrin (1:500, Cell Signaling). After that, they were incubated with HRP-conjugated secondary antibodies for an additional 1 h at room temperature. Equal protein loading of the samples was verified by staining anti-tubulin (Santa Cruz Biotechnology), anti-GAPDH (Cell Signaling, Danvers, MA, USA) or β-actin (1:5000, Santa Cruz Biotechnology). The membranes were developed using ECL (Millipore, Billerica, MA, USA). Gel documentation and signal quantification were made using the Bio-Image Analysis of Molecular Imager ChemiDoc XRS System (Bio-Rad, Richmond, CA, USA).

### Immunofluorescence

Immunolabeling was performed in 5 μm thick sections using a primary antibody to dystrophin (1:200, Santa Cruz Biotechnology) (n = 3-5/group). Secondary antibody fluorescein-conjugated anti-rabbit IgG (Vector Laboratories Inc., Burlingame, CA, USA), diluted 1:200, was used. F-actin filaments were stained with Alexa Fluor 594 conjugated phalloidin (Invitrogen-GIBCO), and DNA was stained with DAPI. The images were analyzed with a Leica DM 6000 M microscope equipped with a Leica AF6000 Deconvolution System (Leica Microsystems).

### Statistical analysis

All data are presented as the mean±SD. Multiple comparisons were made using a one-way analysis of variance (ANOVA) followed by Tukey’s post-test. Differences were considered statistically significant when p<0.05.

## Results

### Echocardiography

**Fig 1:**
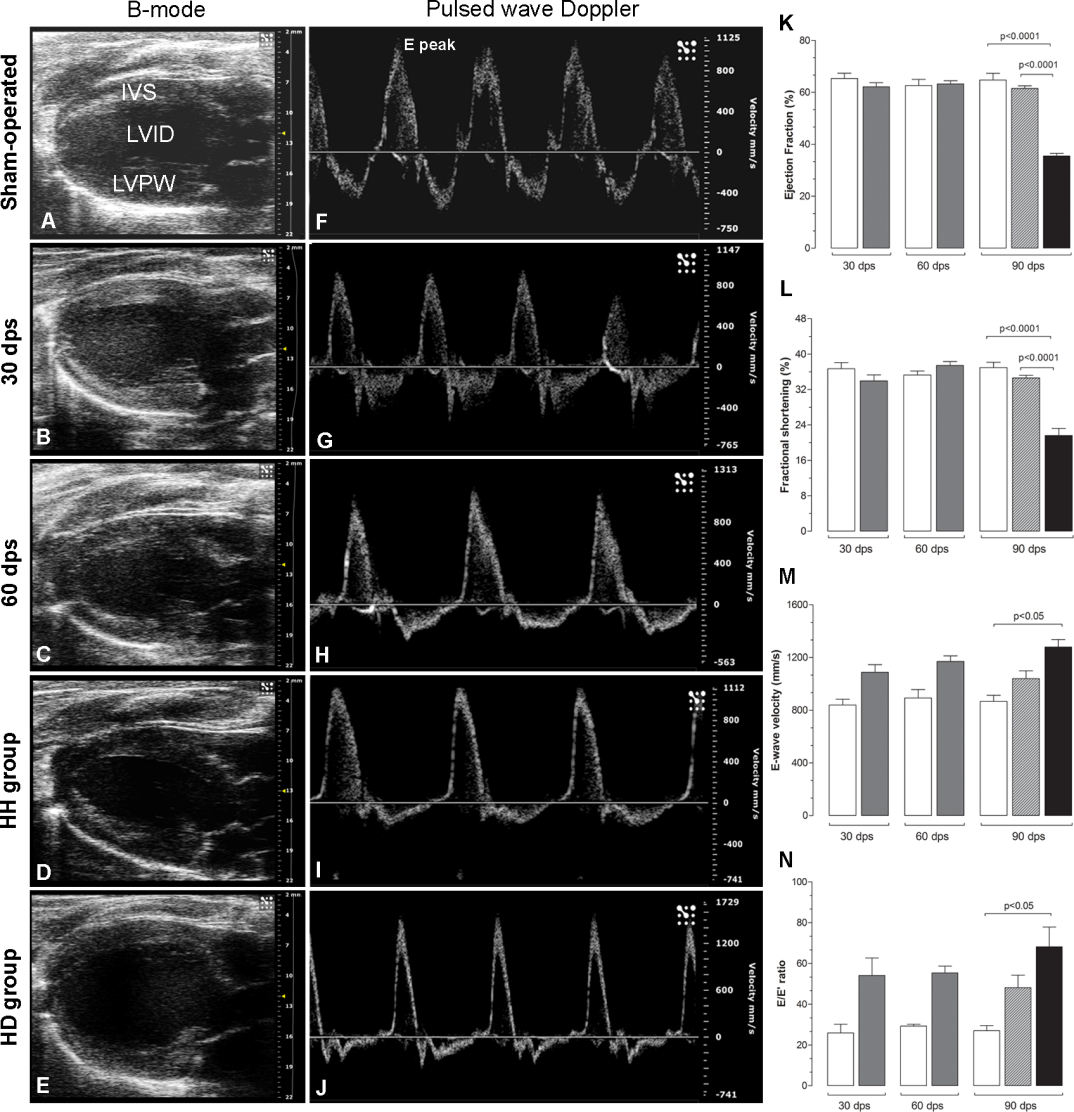
Representative images showing the B-mode long-axis ultrasound (A-E) and pulsed-wave Doppler (F-J) from sham-operated, 30, 60 and 90 dps (HH and HD groups). The B-mode images represent hearts in diastole. Pulsed-wave Doppler shows E peaks in different velocities. The graphs represent data from 3–6 cycles per animal (n = 10-15/group) of the ejection fraction, fractional shortening, E wave velocity and E/E’ ratio. IVS = interventricular septum; LVID = left ventricle internal diameter; LVPW = left ventricle posterior wall. White bars = sham-operated, gray bars = operated; gray bar with stripes = HH group; and black bars = HD group. The values represent the means ± SD.

Table 1 shows changes in the echocardiographic parameters. It was previously shown [25] that after 90 dps the operated animals could be subdivided into two groups, wherein 70% showed hypertrophic hearts (HH) and 30% hypertrophic+dilated hearts (HD). Increased left ventricle wall thickness was observed since 30 dps in IVS and LVPW in both systole and diastole. Alteration in the LVID was observed only at 90 dps in the HD group and increases of 51% and 28% were observed in the systole and diastole, respectively (Table 1; Fig 1A–1E). The systolic function at 30 and 60 dps in the operated groups was not different from the sham-operated group. The findings in HH group were similar to that observed at 30 and 60 dps. However, striking decrease was observed in the systolic function in the HD group: 45% in the EF and 41.5% in the FS (Table 1; Fig 1K and 1L). Concerning to diastolic parameters, there is no difference between sham-operated and operated at 30 and 60 dps and HH group, although increased values were observed. But, in the HD group, the mitral E-wave velocity increased 47%, and E/E' ratio increased 60% (Table 1; Fig 1F–1J, 1M and 1N). The heart rate did not change in sham and operated groups during all the study (Table 1).

**Table 1:** Echocardiographic parameters.

	30d		60d		90d		
	Sham	Operated	Sham	Operated	Sham	HH	HD
IVS systole (mm)	1.81±0.25	2.28±0.39*	2.03±0.31	2.79±0.31***	2.13±0.37	2.72±0.38**	2.85±0.39***
IVS diastole (mm)	1.37±0.12	1.56±0.21*	1.50±0.19	1.93±0.23***	1.48±0.13	1.89±0.26***	1.96±0.26***
LVID systole (mm)	4.87±0.61	5.45±0.63	5.17±0.65	5.56±0.77	5.40±0.55	6.04±0.26^###^	8.18±1.06***
LVID diastole (mm)	7.69±0.73	8.12±0.75	7.80±0.65	8.80±0.90	8.26±0.35	9.10±0.95^###^	10.57±0.88***
LVPW systole (mm)	2.27±0.25	2.69±0.39*	2.37±0.31	3.10±0.39***	2.49±0.11	3.11±0.39***	2.71±0.23
LVPW diastole (mm)	1.74±0.24	1.91±0.24*	1.76±0.23	2.15±0.20**	1.75±0.13	2.11±0.25**	1.98±0.33
EF (%)	65.34±5.77	62.17±5.85	62.62±5.82	63.26±5.42	67.73±7.34	61.46±4.94^###^	35.46±3.29***
FS (%)	36.70±3.88	33.95±5.00	35.28±2.47	37.44±3.81	36.97±3.38	34.60±2.78^###^	21.60±5.34***
Heart rate (bpm)	334.4±50.95	359.9±13.58	329.0±10.27	342.2±21.41	323.5±25.18	325.9±19.99	353.6±22.79
Mitral E-wave velocity (mm/s)	839±99	1087±233	893±142	1169±242	866±134	1039±156	1279±127*
E/E’ ratio	25.89±7.34	54.09±14.91	29.22±1.58	55.27±15.49	26.99±5.47	48.10±14.94	68.14±16.78**

Values are mean ± SD

*p<0.05 versus sham

**p<0.01 versus sham

***p<0.001 versus sham

###p<0.001 HH versus HD.

### Blood pressure

**Fig 2:**
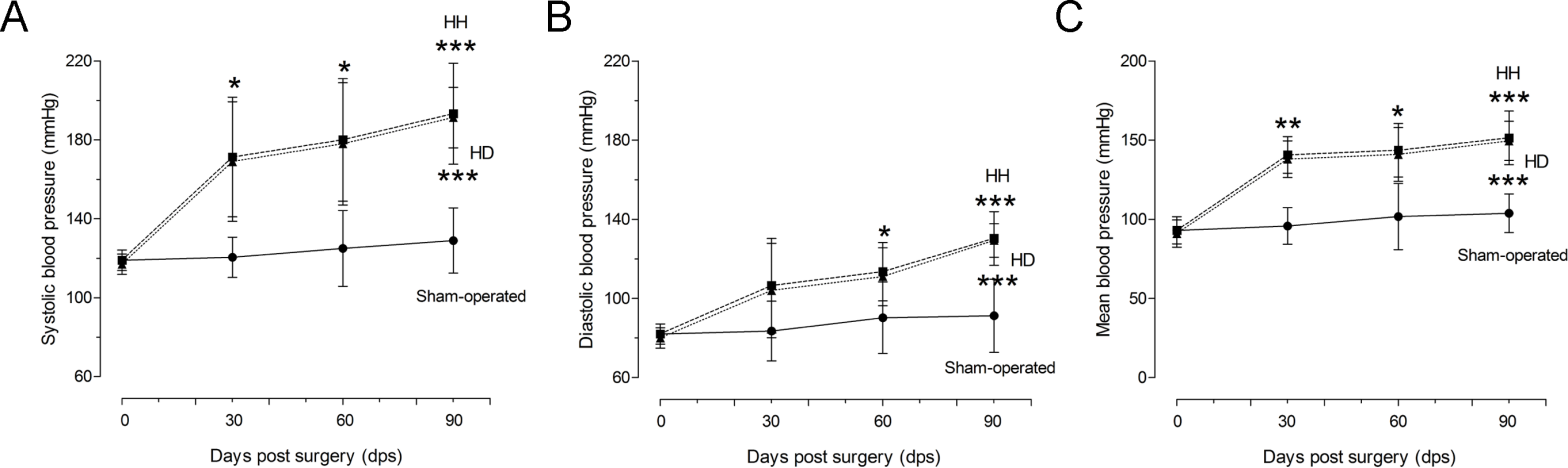
Systolic, diastolic and mean blood pressure. Graphs show data from the systolic, diastolic and mean blood pressure from sham-operated and operated at 30, 60 and 90 dps (HH and HD groups), n = 6-8/group.

To verify whether the increased blood pressure had an additional role in the results, the blood pressure was evaluated. At 30 dps, the systolic blood pressure increased 42% in the operated group (171.3±30.30 mmHg, p<0.05) compared with the sham-operated group (120.5±10.21 mmHg). The increase in diastolic blood pressure was not significant (106.5±23.87 mmHg) compared with the sham-operated group (83.50±15.11 mmHg) and an increase of 47% was observed for the mean blood pressure (140.6±26.41 mmHg, p<0.01) compared with the sham-operated group (95.8±11.56 mmHg) (Fig 2A).

After 60 dps, the increase in the systolic blood pressure was 44% in the operated group (180±31.05 mmHg, p<0.05) compared with the sham-operated group (125.0±19.26 mmHg); the increase in diastolic blood pressure was 29% (113.5±14.69 mmHg, p<0.05) compared to the values of sham-operated group (90.25±18.06 mmHg) and an increase of 41% was observed for the mean blood pressure (143.6±16.95 mmHg, p<0.05) compared with the sham-operated group (101.7±20.85 mmHg) (Fig 2B).

At the end of 90 dps, the increase in the systolic pressure was 50% in HH group (193.3.0±25.56 mmHg, p<0.001) and 48% in HD group (191.3±15.30 mmHg, p<0.001) compared with the sham-operated group (129.0±16.53 mmHg). The increase in the diastolic blood pressure was 43% in HH group (130.3±13.55 mmHg, p<0.001) and 42% in HD group (129.3±8.40 mmHg, p<0.001) compared with the sham-operated group (91.25±18.49 mmHg). An increase of 46% in the HH group (151.4±16.95 mmHg, p<0.001) and 44% in HD group (149.5±12.4 mmHg, p<0.001) in the mean blood pressure was observed compared to the sham-operated group (103.8±12.15 mmHg) (Fig 2C). Here we showed that there is no difference in the blood pressure levels between the HH and HD groups.

### Animal growth and heart morphology

The body weight at 30, 60 and 90 dps was not different between operated and sham-operated groups. The mean heart weight of rats from the operated group was 34% increased at 30 dps, 39% increased at 60 dps, 40% increased in HH group and 76% in HD group at 90 dps when compared with respective sham-operated animals (Table 2). The heart/body weight ratio increased 47% at 30 dps, 38% at 60 dps, 55% in the HH group and 76% in the HD group when compared with respective sham-operated animals. The myocyte diameter increased 11% at 30 dps, 25% at 60 dps, 22% at 90 dps in the HH group and 20% in the HD group when compared with sham-operated animals. Concerning to the interstitial collagen content, significant increases were observed since 30 dps: 71% at 30 dps, 69% at 60 dps, 81% in the HH group and 87% in the HD group at 90 dps when compared to sham-operated animals (Table 2).

**Table 2:** Heart weight, heart/body weight ratio, myocyte diameter and interstitial collagen.

	Heart weight (g)		Heart/body weight ratio (g/Kg)		Myocyte diameter (μm)		Interstitial Collagen (%)	
	Sham	Operated	Sham	Operated	Sham	Operated	Sham	Operated
30d	1.33±0.11	2.00±0.41**	3.25±0.20	4.74±0.40***	10.2±0.24	11.3±0.58**	1.73±0.45	2.96±0.72*
60d	1.71±0.08	2.38±0.29**	3.04±0.20	4.20±0.59**	10.1±0.54	12.6±0.36**	2.07±0.54	3.50±0.72**
90d HH	1.78±0.16	2.66±0.12***	2.99±0.36	4.65±0.44***	10.6±0.73	12.89±0.53**	1.97±0.59	3.53±0.50**
90d HD	1.78±0.16	3.06±0.11***#	2.99±0.36	5.26±0.36***^¶^	10.6±0.73	12.73±0.24**	1.97±0.59	3.69±0.59***

Values are mean±SD

*p<0.05

**p<0.01

***p<0.001 versus sham

#p<0.0001 HD versus HH

¶ p<0.05 HD versus HH.

### Western blotting and immunofluorescence

**Fig 3:**
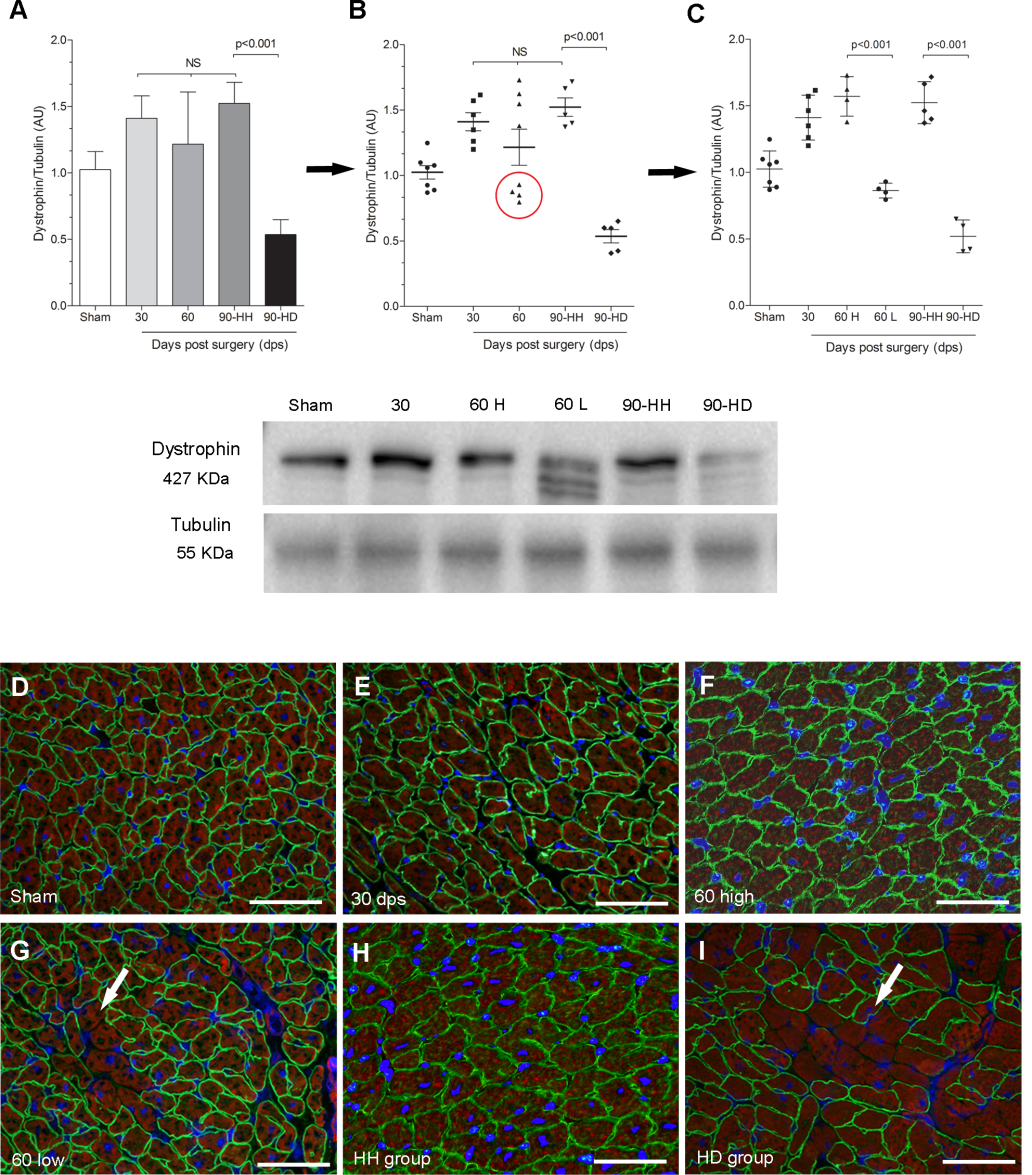
Western blotting and immunofluorescence for dystrophin. Graphs represent data from Western blotting quantification. Immunofluorescent detection of dystrophin (green fluorescence), actin (red fluorescence) and nuclei (blue fluorescence). Bars = 50 microns, n = 5-8/group.

By Western blot quantification, the mean levels of dystrophin expression in the left ventricle at 30 dps (1.41±0.17, Arbitrary Unit—AU) and 60 dps (1.22±0.40 AU) were not different from sham-operated group (1.02±0.14 AU, Fig 3A). A slight increase in the dystrophin expression was observed in the HH group (1.52±0.15 AU, p<0.05) and a marked reduction was observed in the HD group (0.52±0.12 AU, p<0.001), representing a decrease of 50% (Fig 3A). When the mean value is observed in 30 dps, 60 dps and HH group, there is no difference among them (Fig 3A). However, when data are displayed in a scatter plot graph where each data point is displayed individually on the graph, an important finding is highlighted: some animals from the 60 dps group displayed reduction of dystrophin expression (Fig 3B, red circle). This subgroup, named 60 dps Low, showed a reduction of 45% (0.86±0.05 AU, p<0.001) in the dystrophin expression when compared to the other animals from 60 dps group, named 60 dps High (1.57±0.15 AU) (Fig 3C).

The immunofluorescent detection of dystrophin demarcates the cellular perimeter in a uniform pattern as a continuous rim at the periphery of most cardiomyocytes from sham-operated hearts (Fig 3D, green fluorescence). The actin in the cytoskeleton of the cardiomyocytes was very evident (Fig 3D–3I, red fluorescence). At 30 dps and 60 dps High, the pattern of dystrophin labeling was very similar to that observed in the age-matched sham-operated animals (Fig 3E and 3F, respectively). At 60 dps Low, some blocks of cardiomyocytes exhibiting loss of green fluorescence were the first signals of dystrophin loss (Fig 3G). The merged images of dystrophin and actin clearly delineated the cardiomyocytes and highlighted those cardiomyocytes exhibiting loss of dystrophin signal (Fig 3G and 3I, arrow). At 90 dps, an increased number of foci of dystrophin loss were seen in the HD group. HH group results are similar to those observed at 60 dps High (Fig 3H).

**Fig 4:**
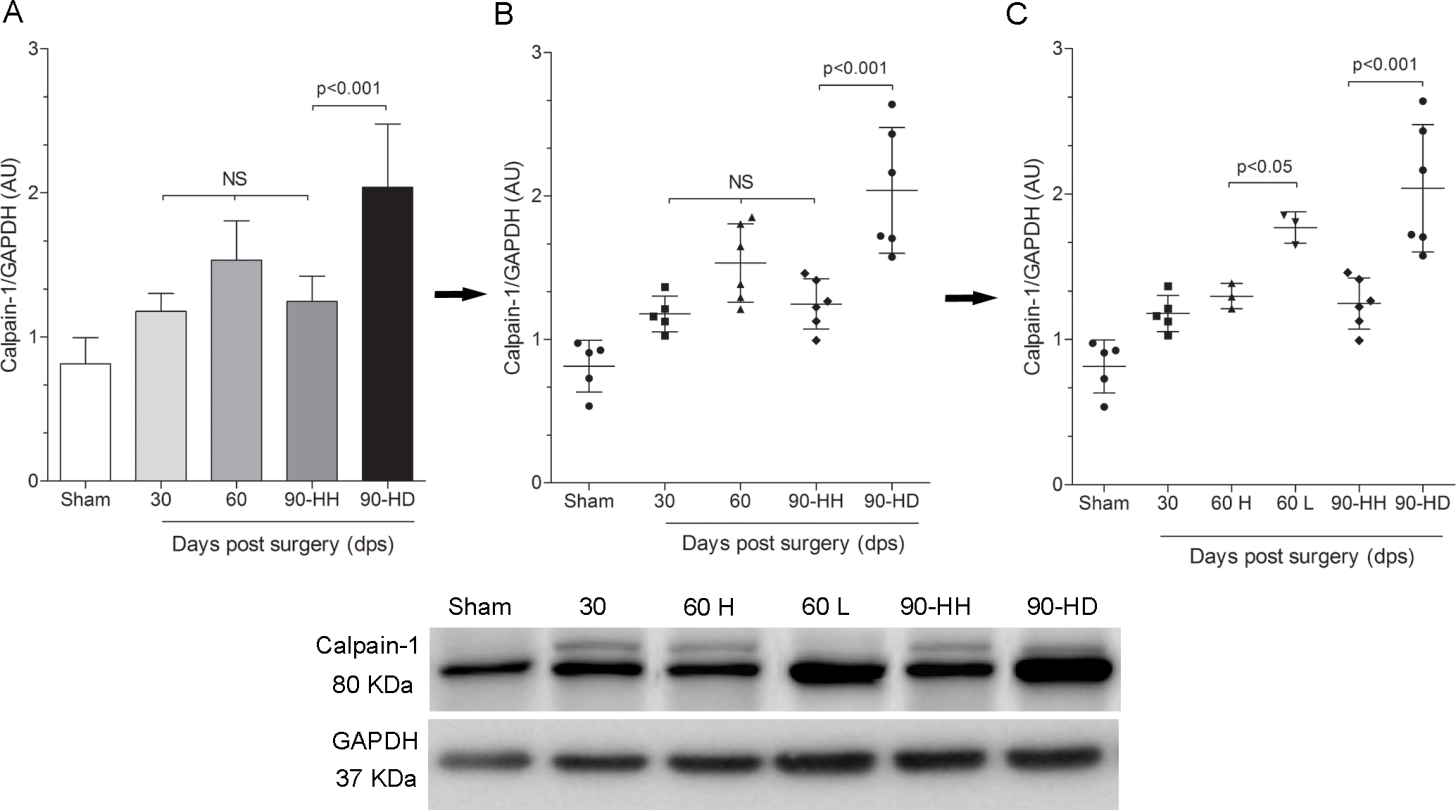
Western blotting for calpain-1. Graphs represent data from Western blotting quantification, n = 5-6/group.

Since calpains have been suggested to be involved in the breakdown of cytoskeletal and sarcomeric proteins during cardiac remodeling, the time-course of calpain-1 protein content was investigated by Western blotting. The protein content of calpain-1 at 30 dps (1.18±0.12 AU), at 60 dps (1.52±0.29 AU) and in the HH group (1.25±0.17 AU) was not different from that observed in the age-matched sham-operated animals (0.81±0.18 AU, Fig 4A). However, a marked increase was observed at 90 dps in the HD group (2.04±0.43 AU) representing an increase of 151% when compared to sham-operated animals (Fig 4A). Following the same line of thought as we made to dystrophin, calpain data were also displayed in a scatter plot graph (Fig 4B) and the same subgroups were identified: 60 dps High (1.30±0.09 AU) and 60 dps Low (1.77±0.11 AU) (Fig 4C). The subgroup 60 dps Low showed an increase of 33% in the calpain expression when compared to 60 dps High (p<0.05).

**Fig 5:**
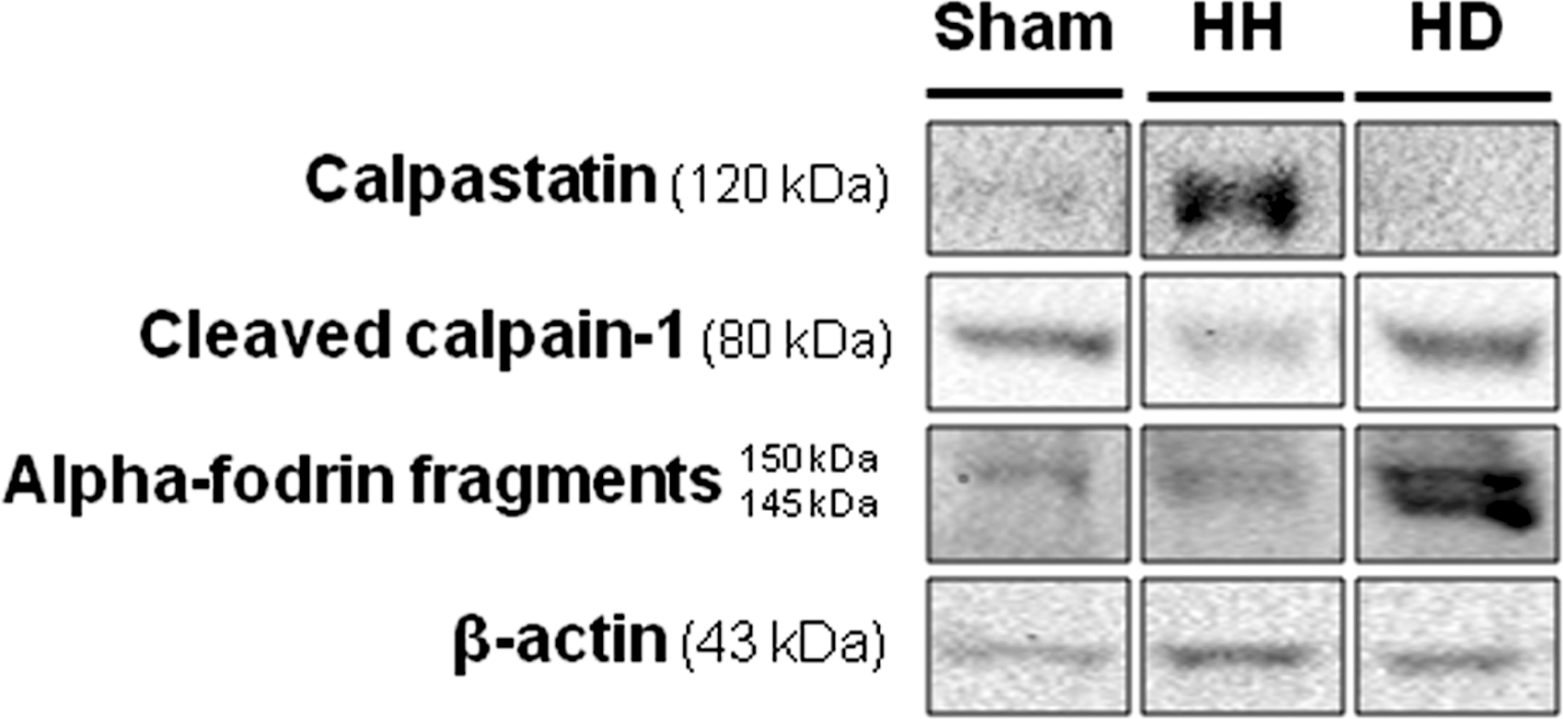
Western blotting analysis for calpastatin, activated calpain-1 and alpha-fodrin. Figure shows representative image of Western blotting in the sham, HH and HD groups, n = 5-6/group.

To further investigate the possible involvement of calpains in the dystrophin degradation, the protein content of calpastatin, a well-known endogenous specific inhibitor of calpains, was investigated. Also, the production of 150- and 145 kDa proteolytic fragments of alpha-fodrin, specific calpain-mediated alpha fodrin degradation products, were investigated. At 90 dps, the protein content of calpastatin was significantly elevated in the HH group, and this effect was associated to low protein content of calpain-1 as well as with low production of the 150- and 145 kDa fragments of alpha-fodrin. However, in the HD group, the protein content of calpastatin was significantly reduced, and at the same time, the protein content of the cleaved calpain-1 and the fragments of alpha-fodrin were significantly increased, suggesting an induction of the calpain-1 activity (Fig 5).

## Discussion

The present study demonstrated that abdominal aorta constriction produced significant hypertension and cardiac hypertrophy from 30 to 90 dps. At 90 dps, 70% of the animals presented cardiac hypertrophy and fibrosis (HH group) associated with preserved dystrophin expression and cardiac function. The remaining 30% presented cardiac hypertrophy+dilated hearts (HD group), decreased dystrophin and increased calpain-1 expression associated with systolic and diastolic dysfunction. At 30 and 60 dps, cardiac hypertrophy and fibrosis were associated with preserved systolic and diastolic cardiac functions. The first signals of dystrophin reduction were observed as early as 60 dps.

The animals from HD and 60 dps High groups presented a significant reduction of dystrophin expression. Dystrophin is an essential cytoskeletal protein in the muscle. The precise physiological role of dystrophin is not fully known although, due to its subsarcolemmal localization, it has been proposed to serve a variety of functions such as providing stability to the membrane and signal transduction [28,29]. Thus, one important function of the dystrophin is to mechanically stabilize the sarcolemmal membrane from shear stress imposed during eccentric muscle contraction [30]. Increased dystrophin mRNA and protein accumulation were observed 7 days after aorta constriction suggesting that dystrophin could play an important role in the maintenance of the sarcolemma integrity under pressure overload [31]. The reduction of dystrophin expression constitutes the structural basis in the hearts of patients with Becker muscular dystrophy and the absence of dystrophin is associated with Duchenne muscular dystrophy. Both genetic disorders are associated with the development of dilated cardiomyopathy [16,32]. Also, dystrophin reduction has been observed in several experimental models of cardiomyopathies, such as post-viral myocarditis caused by Coxsackie virus B [33], myocardial infarction [23], isoproterenol [10,34] and doxorubicin administration [35], septic cardiomyopathy [36] and experimental Chagas disease [37–39]. The importance of dystrophin loss to the development of heart failure has also been demonstrated through the antisense therapeutic dystrophin that restores to almost normal levels the dystrophin expression in the cardiac muscle and prevents the development of cardiac failure in dystrophic *mdx* mice [40].

Significant increases were seen in the cardiomyocytes diameter and collagen content in all experimental groups. Ventricular remodeling is comprised of alterations in both extracellular and intracellular myocardial architecture and fibrosis and cardiomyocyte hypertrophy are classical remodeling parameters. Disproportionate increases in fibrosis and cardiomyocyte decrease the myocardial relaxation, increase stiffness in the ventricular wall, reduce LV compliance and the cardiac function [41–44]. To further investigate the consequences of the cardiac remodeling, the cardiac function was evaluated by echocardiography. Here, serial transthoracic echocardiography and Doppler techniques were used to evaluate LV geometry and systolic and diastolic functions at 30, 60 and 90 dps. Conventional parameters of systolic function including FS and EF remained unchanged at 30 dps and 60 dps groups, suggesting preserved systolic function. However, they were decreased in the HD group, clearly indicating systolic dysfunction. Diastolic function parameters evaluated at 30 and 60 dps groups were not different from control, although increased values were seen. After 90 dps in the HD group, LV dilation and deterioration in all parameters of diastolic filling became apparent. Thus, diastolic dysfunction indicates poor compliance and reduced myocardial relaxation capabilities in this group. Numerous studies have also assessed cardiac structure and function by echocardiography in rats subjected to pressure overload and showed similar results to ours [45–49]. An important limitation of echo studies should be considered: the fusion of the E and A wave. Although E/A ratio is a marker for ventricular relaxation, it could not be obtained in this study since the E and A waves were fused in the rats due to their extremely fast heart rates. It could be argued whether reduced dystrophin expression in the HD group could contribute to the heart failure or this reduction occurs secondary to heart failure? Our results show that the first signals of dystrophin loss were early detected at 60 dps in some animals when systolic and diastolic cardiac function were still preserved. In this way, we can hypothesize that loss of dystrophin occurs before the beginning of cardiac dysfunction. Thus, the animals that early lose dystrophin might be those that could progress to heart failure.

Renin-angiotensin system [50–53] and mechanical stretch [54–58] are activated after abdominal aortic constriction, a well known model of pressure overload induced left ventricular hypertrophy [59–62]. Both stimuli are known to increase intracellular Ca^2+^levels [63–68]. This increase could be responsible for the activation of proteolytic enzymes [18,69,70]. Among these proteolytic enzymes, calpains have an important role in the cardiac remodeling and progression to HF [18,71]. Calpains are a ubiquitous, well-conserved family of Ca^2+^-dependent cysteine proteases, consisting of several tissue-specific isoforms. The main isoforms of calpains in mammalian cells are the μ-calpain (calpain 1) and m-calpain (calpain 2), which are activated in demand of micromolar and millimolar levels of Ca^2+^, respectively. In response to increased levels of cytosolic Ca^2+^ levels, calpains translocate to the cell membrane, where they are activated by autolysis. The cleaved calpains are then released into the cytosol to further cleavage substrates. The calpains activity is inhibited by calpastatin, a well known endogenous inhibitor [71,72]. In this study, we observed increased protein content of calpain-1in the HD group associated with low protein content of calpastatin. To further test the possible activation of calpain, we decided to evaluate another calpain substrate: alpha-fodrin (spectrin). Calpain-mediated digestion of the cytoskeletal protein alpha-fodrin results in two proteolytic fragments of nearly equal electrophoretic mobility (~150 kDa and ~145 kDa). These fragments are the exclusive result of calpain action and can be detected by antibodies against alpha fodrin [73–76]. We showed a significant production of the 150 and 145 kDa proteolytic fragments of alpha-fodrin in the HD group and the low production in the sham and HH groups. Taken together, these data suggest the possible involvement of calpains in the cardiac remodeling observed in the HD group.

In conclusion, some hearts present a distinct molecular pattern at an early stage of the disease; this pattern could provide an opportunity to identify these failure-prone hearts during the development of the cardiac disease. In this study, we showed that decreased expression of dystrophin and increased expression of calpains are coincident and could work as possible therapeutic targets to prevent heart failure as a consequence of cardiac hypertrophy. These changes occur in parallel and this casual relation requires further research. This is a continuing interest of our laboratory.
